# Particle size and cholesterol content of circulating HDL correlate with cardiovascular death in chronic heart failure

**DOI:** 10.1038/s41598-021-82861-6

**Published:** 2021-02-04

**Authors:** Albert Teis, G. Cediel, N. Amigó, J. Julve, J. Aranyó, J. Andrés-Cordón, C. Puig-Jové, E. Castelblanco, F. Gual-Capllonch, E. Ferrer-Sistach, N. Vallejo, G. Juncà, J. López-Ayerbe, M. De Antonio, M. Domingo, E. Santiago-Vacas, P. Codina, D. Mauricio, J. Lupón, Nuria Alonso, A. Bayes-Genis

**Affiliations:** 1 Heart Institute, Cardiology Department, Germans Trias University Hospital, Carretera de Canyet s/n, 08916 Badalona, Barcelona Spain; 2 Department of Medicine, Autonomous University of Barcelona, Barcelona, Spain; 3 Biosfer Teslab, SL, Reus, Spain; 4 Metabolomics Platform, Rovira i Virgili University (URV), Instituto de Investigación Sanitaria Pere Virigili (IISPV), Tarragona, Spain; 5 Center for Biomedical Research on Diabetes and Associated Metabolic Diseases (CIBERDEM), Instituto de Salud Carlos III, Barcelona, Spain; 6 Institut de Recerca de l’Hospital de la Santa Creu i Sant Pau i Institut d’Investigació Biomèdica de l’Hospital de la Santa Creu i Sant Pau, IIB-Sant Pau, Barcelona, Spain; 7 Endocrinology and Nutrition Department, Germans Trias University Hospital, Badalona, Barcelona Spain; 8 Endocrinology and Nutrition Department, Hospital de la Santa Creu i Sant Pau, Barcelona, Spain; 9 Heart Institute, Heart Failure Unit, Germans Trias University Hospital, Badalona, Barcelona Spain; 10 Lleida Biomedical Research Institute’s Dr. Pifarré Foundation (IRBLleida), University of Lleida, Lleida, Spain; 11 Endocrinology and Nutrition Department, Heart Failure Unit, Hospital Universitari Germans Trias i Pujol, Carretera de Canyet s/n, 08916 Badalona, Barcelona Spain; 12 Centre for Biomedical Research on cardiovascular Diseases (CIBERCV), Instituto de Salud Carlos III, Barcelona, Spain

**Keywords:** Biomarkers, Prognostic markers, Heart failure

## Abstract

Evidence regarding any association of HDL-particle (HDL-P) derangements and HDL-cholesterol content with cardiovascular (CV) death in chronic heart failure (HF) is lacking. To investigate the prognostic value of HDL-P size (HDL-Sz) and the number of cholesterol molecules per HDL-P for CV death in HF patients. Outpatient chronic HF patients were enrolled. Baseline HDL-P number, subfractions and HDL-Sz were measured using 1H-NMR spectroscopy. The HDL-C/P ratio was calculated as HDL-cholesterol over HDL-P. Endpoint was CV death, with non-CV death as the competing event. 422 patients were included and followed-up during a median of 4.1 (0–8) years. CV death occurred in 120 (30.5%) patients. Mean HDL-Sz was higher in CV dead as compared with survivors (8.39 nm vs. 8.31 nm, p < 0.001). This change in size was due to a reduction in the percentage of small HDL-P (54.6% vs. 60% for CV-death vs. alive; p < 0.001). HDL-C/P ratio was higher in the CV-death group (51.0 vs. 48.3, p < 0.001). HDL-Sz and HDL-C/P ratio were significantly associated with CV death after multivariable regression analysis (HR 1.22 [95% CI 1.01–1.47], p = 0.041 and HR 1.04 [95% CI 1.01–1.07], p = 0.008 respectively). HDL-Sz and HDL-C/P ratio are independent predictors of CV death in chronic HF patients.

## Introduction

Epidemiological and clinical studies have consistently demonstrated that lower levels of high-density lipoprotein (HDL) cholesterol (HDL-C) are independently associated with coronary heart disease (CHD)^1,2^. However, attempts to reduce cardiovascular (CV) risk by increasing circulating levels of HDL-C have been unsuccessful^3–5^. As a consequence, HDL cardioprotective functions rather than HDL-C quantity has been a new focus of interest.

In the last decade, several studies evaluated the relationship of HDL particles (HDL-P) and their subfractions to CV risk. The pool of circulating HDL is comprised of a heterogeneous mixture of lipoprotein particles and it has been pointed out that small, dense, protein-rich HDLs appear to display potent atheroprotective properties across the HDL subpopulation spectrum, which can be attributed to specific clusters of proteins and lipids^6^. Several authors demonstrated that HDL-P levels have a better correlation with coronary heart disease, coronary heart disease related mortality, coronary artery disease events, and atherosclerosis progression than do the classical lipid profile components such as total cholesterol, triglycerides, or low-density lipoprotein (LDL) cholesterol and HDL-C alone^7–16^. When focusing on HDL subfractions, mainly reduced levels of small HDL-P were associated with CV outcomes^9,17–21^. However, only a few studies have been published analyzing HDL-P and its subfractions in heart failure (HF) patients. A low concentration of small HDL-P has been associated with 3-month all-cause mortality in acute HF patients^22,23^. A recently published report describe an inverse gradation of small HDL-P levels across no-HF, HF with preserved, and HF with reduced ejection fraction patients and its correlation with all-cause mortality and coronary artery disease events in a catheterization lab-based population^24^.

Moreover, the cholesterol content per particle has also emerged as a potential CV marker. As estimated by the ratio of HDL-C to HDL-P (HDL-C/P ratio), it was associated with increased progression of carotid atherosclerosis in a community-based population^16^.

Despite these studies, to date no data has been reported regarding the association of HDL-P concentration, HDL size, HDL-P subfraction distribution, or the HDL-C/P ratio with CV death in chronic HF. Here we investigate the prognostic value of HDL-P, mean normalized HDL size (HDL-Sz), and the HDL-C/P ratio for CV death in patients with ambulatory chronic HF.

## Material and methods

### Study population and outcomes

Ambulatory patients referred to a structured multidisciplinary HF unit of a tertiary university hospital, with an available baseline blood sample, were included from May 2006 to April 2014. Patients were considered for the study regardless of HF etiology. All patients were seen regularly in follow-up visits at the HF unit. The clinical evaluation and treatment of patients were directed by the responsible physician according to a unified protocol, medical criteria, and current clinical practice guidelines^25^. Baseline blood samples were obtained between 09:00 h and 12:00 h and were stored at − 80 °C without previous freeze–thaw cycles. Clinical echocardiograms were also performed at baseline by imaging expert cardiologists in dedicated cardiac echocardiographers (Philips iE33; Philips, The Netherlands) as part of routine clinical practice. Assessments of left ventricular dimensions and function were performed as recommended in guidelines^26,27^. Follow-up visits included a minimum of 1 visit with a nurse every 3 months and 1 visit with a physician every 6 months. The vital status and cause of death (if present) were checked every 3 months. If a patient did not come to a scheduled visit, telephone contact with the patient or patient’s relatives was attempted. If contact was not possible and death was not certified by clinical records from other hospital wards, the emergency department, or general practitioners, vital status was checked from registries of the Spanish Health Systems. Cause of death was adjudicated by the physicians once all available records were reviewed.

The primary endpoint of the study was CV death, defined as mortality due to HF, myocardial infarction, sudden cardiac death, stroke, or secondary to a cardiovascular procedure. Death by unknown cause was an exclusion criteria for the study.

During the baseline visit, patients provided written informed consent for the study. The Local Ethics Committee of Hospital Germans Trias approved the study (code: EO 10-076). The study was performed in compliance with the law protecting personal data, in accordance with the international guidelines on clinical investigation of the World Medical Association’s Declaration of Helsinki^28^.

### Lipoprotein analysis by ^1^H nuclear magnetic resonance spectroscopy

Serum concentrations of triglyceride, cholesterol, HDL-C, the three subclasses (large, medium and small) of HDL particle concentration, and HDL-Sz were assessed at baseline using a novel advanced lipoprotein test based on 2D diffusion-ordered ^1^H nuclear magnetic resonance (NMR) spectroscopy. A 250-μL aliquot of serum from each patient was shipped on dry ice to Biosfer Teslab (Reus, Spain) for lipoprotein analysis using the Liposcale Test. Cut off values for large, medium and small HDL were 9–13 nm, 8.2–9 nm and < 8.2 nm, respectively^29^.The variation coefficients for the number of particle ranged 2% and 4%, and for particle sizes were lower than 0.3%. The total particle count and percentage of total HDL-P constituted by each subfraction were also assessed. The HDL-C/P ratio was calculated as an estimate of cholesterol content of the HDL particles^16^. To calculate the ratio, units of HDL-C were transformed to μmol/L.

### Statistical analysis

Categorical variables are expressed as the absolute number and percentages. Continuous variables are expressed as means ± SD or medians (quartiles Q1–Q3) for normal and non-normal distributions, respectively. Data distributions were assessed with normal Q–Q plots. Differences between groups were determined using the chi-squared test, Student’s *t* test, Mann–Whitney *U* test, Kruskal–Wallis test, and means comparison (ANOVA), as appropriate. To evaluate the association of candidate variables with CV death, univariable and multivariable Cox regression analyses were performed with CV death as the dependent variable. Multivariable analyses using a stepwise selection method (backward elimination, removing all variables with a p value > 0.05) were performed and included the significant covariates from the univariable analysis and other relevant clinical variables (age, sex, body mass index, diabetes, arterial hypertension, vasculopathy, ischemic etiology, New York Heart Association [NYHA] functional class III-IV, estimated glomerular filtration rate [eGFR], hemoglobin, N-terminal prohormone of brain natriuretic peptide [NT-ProBNP], LDL particles, and treatment with angiotensin-converting enzyme inhibitors [ACEi], angiotensin receptor blockers [ARB], beta blockers, statins, and implantable cardioverter-defibrillators [ICD]). A competing risk strategy using the Fine and Gray method was adopted, considering non-CV death as a competing event. All lipid variables were standardized and, accordingly, the resulting hazard ratios reflect a 1-SD change in the given parameter. Survival curves were generated from the full model using tertiles of HDL-Sz and the HDL-C/P ratio. Given the known association of HDL-P with inflammatory status, additional sensitivity analysis was performed, including in the initial model the glycoprotein (Gly) A, Gly-B, and Gly-F levels, which are three validated, composite ^1^H-NMR spectroscopy-derived markers of systemic inflammation^30–33^. Considering that the metabolic profile could change during disease progression, a secondary analysis was performed, according to NYHA functional class. Finally, considering the high prevalence of ischemic etiology in our cohort, the interaction between HDL-Sz and the HDL-C/P ratio with ischemic etiology was assessed, and found not significant in any case (p = 0.741 and p = 0.809, respectively). Moreover, the interaction between HDL-Sz and the HDL-C/P ratio with HF and preserved or reduced ejection fraction was assessed, being not significant in any case (p = 0.818 and p = 0.911, respectively).

Statistical analyses were performed using STATA V.13.0 (College Station, Texas, USA). A two-sided p < 0.05 was considered significant.

## Results

### Clinical characteristics

A total of 422 patients were included. The baseline characteristics of the subjects are listed in Table 1. The main causes of HF included ischemic heart disease (48.1%), dilated cardiomyopathy (15.9%), and hypertensive cardiomyopathy (10.2%) (Table 2). As compared with survivors patients, CV death patients were older (73.6 vs. 61.4 years; p < 0.001), with higher prevalence of hypertension, (76.7% vs. 59%; p = 0.001), peripheral vasculopathy (25% vs. 9%; p < 0.001), ischemic heart disease (65% vs. 36.5%; p = 0.001), and atrial fibrillation (32.5% vs. 16.6%; p = 0.003). They also presented with worse baseline NYHA functional class (NYHA III-IV 33.3% vs. 13.1%; p < 0.001), eGFR (45.9 ± 25.5 vs. 75.3 ± 36.0 mL/min/1.73 m^2^; p < 0.001) and higher NT-proBNP levels (3949 [1765–7228] vs. 1103 [442–2544] pg/mL; p < 0.001). No differences in left ventricular ejection fraction were observed among groups. Interestingly, CV death patients received a lower proportion of beta blockers (88.3% vs. 95%; p = 0.020), ACEi or ARB (76.7% vs. 91.9%; p < 0.001), or mineralcorticoid receptor antagonists (59.2% vs. 70.3%; p = 0.037), but required more loop diuretics (97.5% vs. 82.9%; p < 0.001). Statins were used slightly more in the living population (74.2% vs. 82.8%; p = 0.030). Moreover, less ICD therapy was used in the CV death patients (10.0% vs. 24.7%; p < 0.001).

**Table 1:** Baseline characteristics of patients.

	Overall	Non-survivors		Survivors	P
		CV death	Non-CV death		
	n = 422	n = 120	n = 80	n = 222	
Age, years	67.2 ± 13.4	73.6 ± 10.1	73.7 ± 9.9	61.4 ± 13.4	< 0.001
Male	312 (72.7)	86 (71.7)	60 (75)	161 (72.5)	0.869
Hypertension	284 (67.3)	92 (76.7)	61 (76.3)	131 (59)	0.001
Diabetes	191 (45.3)	61 (50.8)	41 (51.2)	89 (40)	0.080
Hypercholesterolemia	275 (65.2)	79 (65.8)	56 (70)	140 (63)	0.525
COPD	66 (15.6)	23 (19.2)	17 (21.3)	26 (11.7)	0.060
Vasculopathy	66 (15.6)	30 (25.0)	16 (20)	20 (9)	< 0.001
NYHA Class III–IV	91 (21.6)	40 (33.3)	22 (27.5)	29 (13.1)	< 0.001
BMI	27.38 ± 4.8	26.88 ± 5.00	27.11 ± 4.42	27.73 ± 4.83	0.250
HR, bpm	69.8 ± 14.8	72.8 ± 14.7	69.6 ± 15.0	68.2 ± 14.7	0.025
Atrial fibrillation	96 (22.7)	39 (32.5)	20 (25)	37 (16.6)	0.003
LBBB	53 (12.6)	17 (14.2)	6 (7.5)	30 (13.5)	0.312
LVEF, %	35.6 ± 14.2	36.1 ± 15.5	37.5 ± 14.7	34.6 ± 13.2	0.270
LVEDd, mm	59.9 ± 9.0	59.3 ± 9.6	58.7 ± 8.5	60.5 ± 8.9	0.227
**Blood tests**					
Hb, g/dL	12.7 ± 1.8	12.1 ± 1.7	12.2 ± 1.5	13.2 ± 1.8	< 0.001
Glucose, mg/dL*	120.15 ± 3.0	118.05 ± 5.7	126.11 ± 5.4	119.1 ± 4.4	0.490
Sodium, mmol/L	137.9 ± 3.5	137.7 ± 3.94	138.1 ± 3.1	138.0 ± 3.4	0.706
Potassium, mmol/L	4.24 ± 0.03	4.28 ± 0.05	4.21 ± 0.06	4.23 ± 0.03	0.587
eGFR, mL/min/1.73m^2^	62.3 ± 34.8	45.9 ± 25.5	50.9 ± 29.1	75.3 ± 36.0	< 0.001
NT-proBNP, pg/mL [median (Q1-Q3)]	1811 (735–4414)	3949 (1765–7228)	2921 (1027–5074)	1103 (442–2544)	< 0.001
**Treatment at baseline**					
Betablockers	389 (92.2)	106 (88.3)	72 (90)	211 (95)	0.020
ACEi/ARB	360 (85.3)	92 (76.7)	64 (80)	204 (91.9)	< 0.001
MRA	279 (66.1)	71 (59.2)	52 (65)	156 (70.3)	0.037
Loop diuretic	373 (88.4)	117 (97.5)	72 (90)	184 (82.9)	< 0.001
Statins	329 (78)	89 (74.2)	56 (70)	184 (82.8)	0.030
ICD	70 (16.6)	10 (10.0)	3 (3.8)	55 (24.8)	< 0.001
CRT	53 (12.6)	13 (10.8)	5 (6.3)	35 (15.8)	0.071
^**1**^**H-NMR lipid biomarkers**					
Total TG, mg/dL	129.0 ± 54.4	116.9 ± 47.8	131.4 ± 49.4	134.7 ± 58.6	0.014
Total cholesterol, mg/dL	195.2 ± 38.0	184.9 ± 30.5	193.8 ± 37.0	201.3 ± 40.8	0.001
LDL-C, mg/dL	110.6 ± 27.9	103.4 ± 23.2	108.7 ± 26.9	115.3 ± 29.8	0.001
HDL-C, mg/dL	48.6 ± 10.4	49.2 ± 9.9	47.3 ± 9.8	48.7 ± 10.9	0.436
HDL-Sz, nm	8.34 ± 0.14	8.39 ± 0.17	8.36 ± 0.15	8.31 ± 0.11	< 0.001
**HDL particle number, µmol/L (% over total)**					
Total	25.7 ± 5.4 (100%)	25.2 ± 5.1 (100%)	25.1 ± 5.3 (100%)	26.2 ± 5.7 (100%)	0.133
Large	0.3 ± 0.05 (1.2%)	0.3 ± 0.1 (1.2%)	0.3 ± 0.1 (1.2%)	0.3 ± 0.1 (1.2%)	0.743
Medium	10.2 ± 2.0 (41,0%)	10.7 ± 1.9 (44.2%)	10.3 ± 1.9 (42.4%)	9.9 ± 2.0 (38.8%)	0.002
Small	15.2 ± 4.9 (57,7%)	14.2 ± 5.0 (54.6%)	14.5 ± 4.9 (56.4%)	16.0 ± 4.7 (60.0%)	0.002
HDL-C/P ratio	49.2 ± 5.9	51.0 ± 6.9	49.2 ± 6.6	48.3 ± 4.6	< 0.001

Results expressed as mean ± SD for quantitative variables and n (%) for categorical unless otherwise indicated.

*COPD* chronic obstructive pulmonary disease, *BMI* body mass index, *BSA* body surface area by Mosteller formula, *HR* heart rate, *AF* atrial fibrillation, *LBBB* left bundle branch block, *LVEF* left ventricular ejection fraction, *LVEDd* left ventricular end-diastolic diameter, *Hb* hemoglobin, *eGFR* estimated glomerular filtration rate calculated by Cockoft–Gault formula, *ACEi* Angiotensin Converting Enzyme Inhibitor, *ARB* Angiotensin II receptor blockers, *MRA* mineralcorticoid receptors antagonists, *ICD* Implantable Cardiac Defibrillator, *CRT* Cardiac Resyncronization, *NMR* nuclear magnetic resonance, *TG* triglycerides, *HDL-Sz* mean normalized HDL particle size, *LDL-C* LDL cholesterol, *HDL-C* HDL cholesterol.

*Basal Glucose levels only available in 336 patients (in 101 CV death, 66 non-CV death and in 169 survivors).

**Table 2:** Etiology of heart failure.

	Total	Death		Alive	P value
		CV death	Non-CV death		
	n = 422	n = 120	n = 80	n = 222	
Ischemic	203 (48.1)	78 (65.0)	44 (55)	81 (36.5)	< 0.001
DCM	67 (15.9)	11 (9.2)	9 (11.3)	47 (21.2)	
Hypertensive	43 (10.2)	10 (8.3)	10 (12.5)	23 (10.4)	
Alcoholic	22 (5.2)	0	3 (3.8)	19 (8.5)	
Drugs/chemotherapy	14 (3.3)	1 (0.8)	3 (3.8)	10 (4.5)	
Valve disease	33 (7.8)	14 (11.7)	6 (7.5)	13 (5.8)	
Other	21 (5.0)	3 (2.5)	5 (6.3)	13 (5.8)	
HCM	8 (1.9)	2 (1.7)	0	6 (2.7)	
Non-compaction	5 (1.2)	0	0	5 (2.2)	
Amyloid	3 (0.7)	1 (0.8)	0	2 (0.9)	
Peripartum	2 (0.5)	0	0	2 (0.9)	
Tachycardiomyopathy	1 (0.2)	0	0	1 (0.4)	

Results expressed as n (%).

*DCM* dilated cardiomyopathy, *HCM* hypertrophic cardiomyopathy.

### NMR spectroscopy lipoprotein profiling

**Figure 1:**
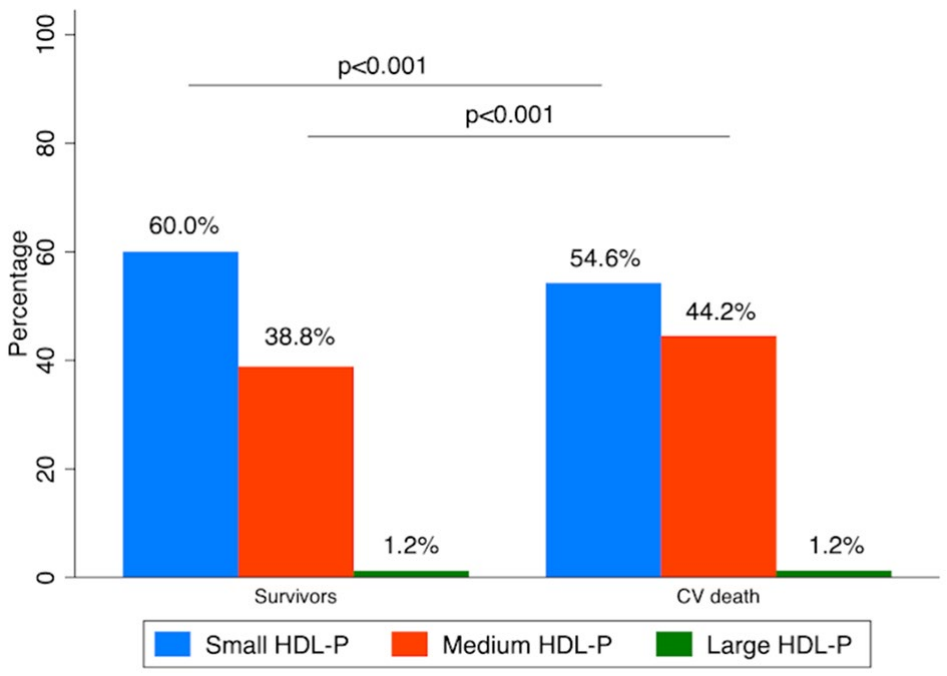
Distribution of HDL particles according to their size in survivors and patients who suffered cardiovascular death. *HDL-P* HDL particle, *CV* cardiovascular.

Serum concentrations of total triglycerides, total cholesterol, HDL-Sz, HDL subfraction particle numbers, and the calculated HDL-C/P ratio are listed in Table 1. Total cholesterol and LDL cholesterol were lower in the CV death group in comparison with the living patients (184.9 ± 30.5 vs. 201.3 ± 40.8 and 103.4 ± 23.2 vs. 115.3 ± 29.8 respectively; both p = 0.001). No differences in HDL-C levels or in the number of HDL-P across groups were observed, but HDL-Sz was bigger in the CV death group than in the living patients (8.39 ± 0.17 nm vs. 8.31 ± 0.11 nm, p < 0.001). This change in size was mainly produced by a decrease in the percentage of small HDL-P (54.6% and 60% for CV death and living patients, respectively; p < 0.001), with a consequent proportional variation in the percentage of medium HDL-P (Fig. 1). Additionally, the HDL-C/P ratio, an estimator of the degree of cholesterol content of the HDL particles, was higher in CV death patients (51.0 ± 6.9 vs. 48.3 ± 4.6 for CV death and survivors respectively; p < 0.001).

Correlations of HDL-Sz and the HDL-C/P ratio with HDL-C and levels of Gly-A, Gly-B, and Gly-F were assessed. We found weak but statistically significant correlations of HDL-C with HDL-Sz (r^2^ = − 0.28; p < 0.01) and the HDL-C/P ratio (r^2^ = 0.24; p < 0.01). The correlation of study variables with markers of systemic inflammation revealed a weak but statistically significant inverse correlation of HDL-Sz with Gly-A (r^2^ = − 0.28, p < 0.01), Gly-B (r^2^ = − 0.08, p = 0.09), and Gly-F (r^2^ = − 0.35, p < 0.01). For the HDL-C/P ratio, a modest inverse correlation was found with Gly-A (r^2^ = − 0.64, p < 0.01) and Gly-F (r^2^ = − 0.67, p < 0.01), and a weak inverse correlation with Gly-B (r^2^ = − 0.26, p = 0.07).

### Association of HDL-Sz and HDL-C/P ratio with cardiovascular death

During a median follow-up of 4.1 (0–8) years, the primary endpoint occurred in 120 patients (30.5%). Main causes of CV death were HF (59.2%), sudden cardiac death (18.3%), myocardial infarction (9.2%), and stroke (1.7%). Other causes of death corresponded to 11.6% of cases.

**Figure 2:**
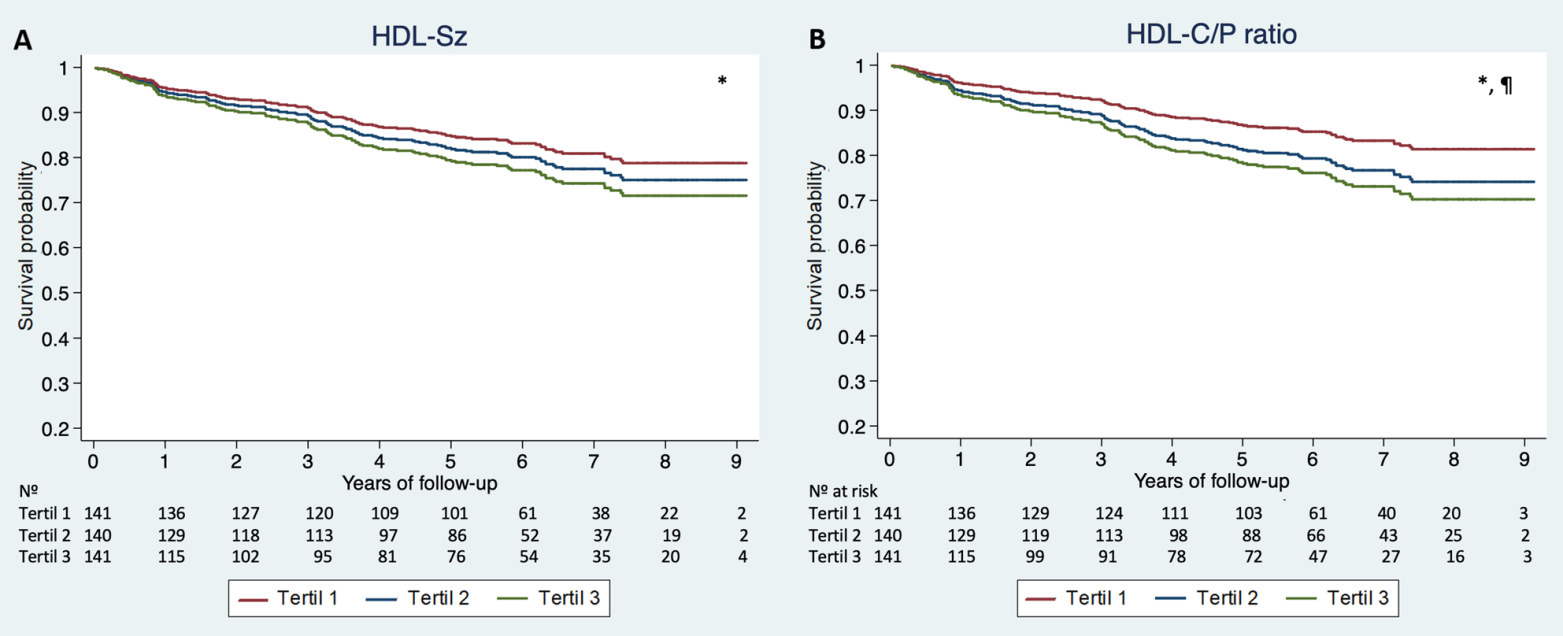
Survival curves showing the probability over time to observe a cardiovascular death across Tertiles of HDL-Sz and HDL-C/P ratio. Survival curves showing the probability over time to observe a cardiovascular death across Tertiles of normalized mean HDL-P size (HDL-Sz) and HDL-C/P ratio, adjusted for age, sex, BMI, diabetes, arterial hypertension, vasculopathy, ischemic etiology, NYHA functional class III–IV, eGFR, Hemoglobin, NT-ProBNP and treatment with ACEi/ARB, Betablockers, statins and LDL-P and ICD. Statistical significance (p < 0.05) denoted as follows: *significant difference in CV death between the highest and lowest tertiles; ^¶^significant difference in CV death between the highest and middle tertiles. *HDL-C/P* HDL cholesterol over HDL particle ratio, *HDL-Sz* normalized mean HDL size.

As shown in Table 3, a multivariable competing-risk regression analysis demonstrated that both HDL-Sz and the HDL-C/P ratio were independent predictors of the occurrence of CV death during follow-up (HR 1.21, 95% CI 1.01–1.47; p = 0.041 and HR 1.04, 95% CI 1.01–1.07; p = 0.008, respectively). Considering the reported biological interaction between HDL function and inflammatory status, we performed an additional sensitivity analysis, adding Gly-A, Gly-B, and Gly-F to the initial model, with consistent results (HR 1.22, 95% CI 1.01–1.47; p = 0.041 and HR 1.04, 95% CI 1.01–1.47; p = 0.008; for HDL-Sz and the HDL-C/P ratio, respectively). Adjusted survival curves were generated using tertiles of HDL-Sz and the HDL-C/P ratio, showing increased risk of CV death over time for the higher tertiles of both variables (Fig. 2).

**Table 3:** Univariable and multivariable Cox Regression Analysis of the associations between HDL-Sz and HDL-C/P ratio with cardiovascular death.

	HDL-Sz		HDL-C/P ratio	
	HR (95% CI)	P value	HR (95% CI)	P value
Unadjusted	1.36 (1.16–1.58)	< 0.001	1.06 (1.03–1.09)	< 0.001
**Multivariable adjustment**				
Model 1	1.21 (1.01–1.47)*	0.041	1.04 (1.01–1.07)*	0.008
Model 2	1.22 (1.01–1.47)*	0.041	1.04 (1.01–1.07)*	0.008

Hazard ratios for HDL-Sz reflect 1 SD change in a given measure.

**Model 1** Adjusted for age, sex, BMI, diabetes, arterial hypertension, vasculopathy, ischemic etiology, NYHA functional class III-IV, eGFR, Hemoglobin, NT-ProBNP, LDL-P and treatment with ACEi/ARB, Betablockers, statins and ICD.

**Model 2** Adjusted for Model 1 plus glycoprotein (Gly)-A, Gly-B and Gly-F.

*Other independent predictors of the outcomes in these models besides HDL-Sz or HDL-C/P were age, ischaemic etiology and NT-proBNP.

**Figure 3:**
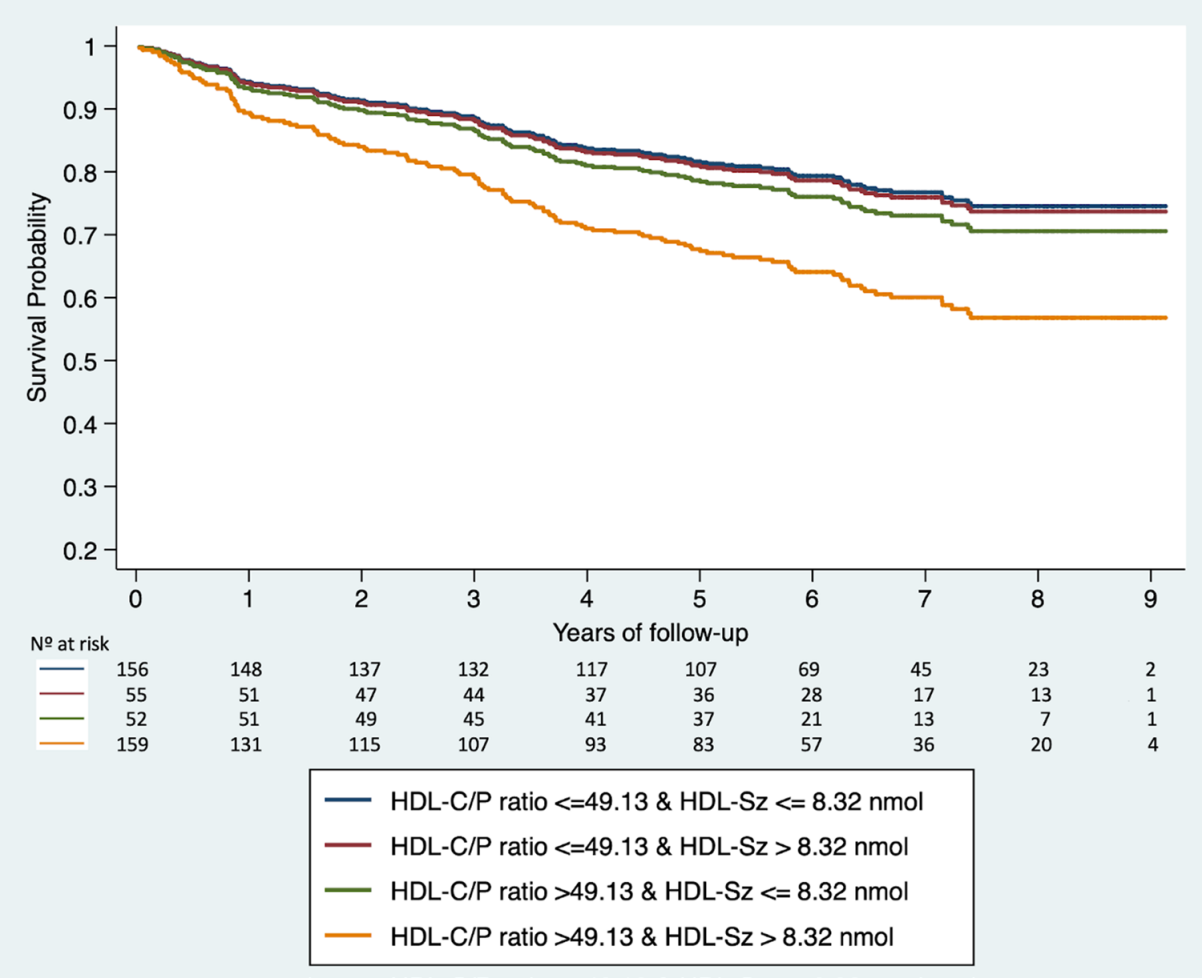
Survival curves showing the probability over time to observe a cardiovascular death according to the HDL-C/P ratio and normalized mean HDL-P size (HDL-Sz). *HDL-C/P ratio* HDL cholesterol over HDL particle ratio, *HDL-Sz* normalized mean HDL particle size.

The dispersion of HDL-Sz and HDL-C/P ratio values was also represented, and the total cohort was divided into four equally sized groups accounting for HDL-Sz and HDL-C/P median values (8.32 nm and 49.13, respectively) (Supplementary material, Fig. S1). Cases with increased HDL-Sz and highly cholesterol-loaded HDL-P were at higher risk of CV death (HR 1.94, 95% CI 1.28–2.96; p = 0.002) (Fig. 3).

Finally, we performed an additional regression analysis according to NYHA functional class evidencing that the association of the variables of interest with the outcome of the study remains only significant in the subgroup of patients with NYHA functional class III-IV and approaching a level of significance for HDL-Sz in patients with NYHA functional class I-II (Supplementary material, Table S1).

## Discussion

In this study, circulating particle concentrations of HDL and its subfractions were quantified using ^1^H-NMR spectroscopy in a real-life chronic out-patient clinic HF cohort with exquisite long-term clinical follow-up of the patients. We observed significant derangements in mean HDL-Sz and the HDL-C/P ratio in those with CV death as compared with survivors after comprehensive multivariable adjustment. Changes in mean HDL-Sz were mainly due to a decrease in the proportion of small HDL-P rather than an increase in large HDL-P. Moreover, the degree of cholesterol content of the HDL, represented by the HDL-C/P ratio, was increased in those CV death patients. We observed that HDL-Sz and elevated levels of cholesterol-overloaded HDL, understood as the HDL cholesterol content per particle (HDL-C/P ratio), were independently associated with the increased risk of CV death in HF patients. Overall, these findings suggest that alterations in the distribution among HDL subfractions and the degree of its cholesterol content are important markers, beyond total cholesterol or HDL-C levels, for CV death in out-patient clinic chronic HF patients.

To date, evidence on association of HDL-P and its subfractions in HF patients is scarce, with no data from real-life chronic out-patient HF population. Potočnjak et al. and Degoricija et al. identified inverse associations of total HDL-P and levels of small HDL-P with very short (3-month) survival in patients hospitalized with acute HF^22,23^. Hunter et al. found an inverse gradient association of HDL-P count and small HDL-P with all-cause mortality and coronary artery disease events in no-HF, HF with preserved and HF with reduced ejection fraction patients in a cath-lab based population^24^. As in those reports, we also found that the size of HDL (HDL-Sz) and percentage of small HDL-P were crucial and associated with adverse events, increasing the importance of this marker in the HF population. However, we did not observe any difference in total HDL-P count between CV death and survivors, contrary to the other groups’ results. One explanation is that our cohort differs from both of their’s, being more representative of real-life chronic HF, with lower overall total HDL-P count across the entire cohort, regardless of its future prognosis. Although the comparison must be cautious because different ^1^H-NMR spectroscopy tests were used, the total HDL-P count in our cohort is also lower as compared with the HF with reduced ejection fraction subgroup of Hunter et al.’s population (25.7 ± 5.4 vs. 27.2 ± 6.2, for our entire cohort vs. Hunter et al.’s cohort). Both groups showed no differences in total cholesterol or HDL-C between alive and dead patients, emphasizing the importance of HDL subfraction analysis over total cholesterol or HDL-C in HF. In our study, no significant differences in serum HDL-C were observed between survivors and CV death patients; however, total cholesterol concentrations were lower in dead patients. One explanation is that our cohort corresponds to a real-life chronic HF population, and CV death patients could present with lower total cholesterol levels due to more severe and evolved HF process. The cholesterol paradox in HF has been reported previously and has been related to cachexia^34,35^. In fact, those with CV death were older, presented with worse NYHA class, higher NT-proBNP levels, more tachycardia, more incidence of atrial fibrillation and required more loop diuretics. As a consequence of their worse clinical status, less beta blocker, ACEi, ARB, or mineralcorticoid receptor antagonist treatment was given. In keeping with this, statins were used slightly less in the CV death population as compared with the survivors. However, statin treatment was included in all the multivariate models, and the association of HDL-Sz and the HDL-C/P ratio with CV death remained significant.

The HDL-C/P ratio as a surrogate of cholesterol content of HDL-P was previously reported by Qi et al.^16^ and later explored by Amigó et al.^36^. The average cholesterol content per particle reflects the HDL particle distribution, being higher in larger HDL particles, as while the size increases linearly, the cholesterol content increases with a cubic relationship. Qi et al. observed that cholesterol-overloaded HDL-P was independently associated with the progression of carotid atherosclerosis in a disease-free population. To our knowledge, the HDL-C/P ratio has not previously been explored as a marker of adverse cardiovascular events in HF patients. Interestingly, as demonstrated in our study, the HDL-C/P ratio is an independent predictor for CV death regardless of the HDL-Sz or HDL-P count in HF. This might suggest that derangements in cholesterol transport of HDL are also critical in the prognosis of chronic HF patients and that this parameter might be used as a biomarker to stratify CV death risk in this population. An anomalous clearance of HDL-C may be one of the explanations for these findings, resulting in a final increase in HDL-C/P ratio despite total HDL-C levels. Given the evidence that medium and large subfractions of HDL-P are not reduced, the overall cholesterol efflux appears not to be significantly affected in chronic HF patients. However, the final pathophysiological mechanism that could explain these findings should be explored in further studies. Finally, we also hypothesized that the decrease in small HDL-P might suggest a decline in the cardioprotective activity of HDL, which is mainly mediated by small-sized HDL^6,37^. However, how these molecular effects can be translated at the clinical level should be confirmed in future investigations.

### Strengths and limitations

The strengths of our study are that it is representative of real-life chronic HF patients, with an exquisite follow-up. Moreover, the relationship of HDL-Sz and HDL-C/P to CV death was not only assessed in a comprehensive multivariate model, but also confirmed after adjustment for additional sensitivity analysis to rule out any confusing effect of HDL-P and its subfractions with any inflammatory status relevant to HF pathobiology.

This study also has some limitations. A selection bias could be present, as patients were included only if a baseline blood sample was available. Although it identifies associations of HDL-Sz and the HDL-C/P ratio with CV death, it does not prove a causal role of these derangements with adverse outcomes. However, the results align with emerging evidence suggesting an association of mainly small HDL-P with events in HF.

Another limitation is that it is a single-center population, and additional clinical investigations regarding the association of HDL-P with CV death in HF should be performed. Although several adjusted multivariate models were used, unmeasured confounders may have had an impact on the results.

Finally, apolipoprotein A levels or other standard inflammatory parameters beyond Glycoprotein A, B and F were not measured. Any interaction of those with the results is unknown.

## Conclusions

In a chronic out-patient-clinic HF population, significant derangements in mean HDL-P size and HDL-C/P ratio were associated with CV death. Changes in mean HDL-Sz were mainly due to a decrease in the proportion of small HDL-P subfraction. Those with higher HDL-Sz were in higher risk for CV death. Moreover, the degree of cholesterol loading of the HDL, represented by the HDL-C/P ratio, was increased in those with CV death. This ratio has not been explored as a marker of adverse cardiovascular events in HF patients to date. These associations were robust, considering the rigorous multivariable adjustment. These data may help refine risk assessment and provide new insights into the complex interaction of HDL and HF pathophysiology. Further investigation is needed to elucidate the mechanisms underlying the observed associations and a better understanding of the role of HDL subfractions and cholesterol content of these particles in chronic HF.

## Supplementary Information

Supplementary Information

## References

[1] The Emerging Risk Factors Collaboration* (2009). Major lipids, apolipoproteins, and risk of vascular disease. JAMA.

[2] Gordon T, Castelli WP, Hjortland MC, Kannel WB, Dawber TR (1977). High density lipoprotein as a protective factor against coronary heart disease. Am. J. Med..

[3] Barter PJ (2007). Effects of torcetrapib in patients at high risk for coronary events. N. Engl. J. Med..

[4] Boden WE (2011). Niacin in patients with low HDL cholesterol levels receiving intensive statin therapy. N. Engl. J. Med..

[5] Schwartz GG (2012). Effects of dalcetrapib in patients with a recent acute coronary syndrome. N. Engl. J. Med..

[6] Camont L, Chapman MJ, Kontush A (2011). Biological activities of HDL subpopulations and their relevance to cardiovascular disease. Trends Mol. Med..

[7] Otvos JD (2006). Low-density lipoprotein and high-density lipoprotein particle subclasses predict coronary events and are favorably changed by Gemfibrozil therapy in the veterans affairs high-density lipoprotein intervention trial. Circulation.

[8] Kuller LH, Grandits G, Cohen JD, Neaton JD, Prineas R (2007). Lipoprotein particles, insulin, adiponectin, C-reactive protein and risk of coronary heart disease among men with metabolic syndrome. Atherosclerosis.

[9] McGarrah RW (2016). High-density lipoprotein subclass measurements improve mortality risk prediction, discrimination and reclassification in a cardiac catheterization cohort. Atherosclerosis.

[10] El Harchaoui K (2009). High-density lipoprotein particle size and concentration and coronary risk. Ann. Intern. Med..

[11] Parish S (2012). Lipids and lipoproteins and risk of different vascular events in the MRC/BHF heart protection study. Circulation.

[12] Mackey RH (2012). High-density lipoprotein cholesterol and particle concentrations, carotid atherosclerosis, and coronary events: MESA (Multi-Ethnic Study of Atherosclerosis). J. Am. Coll. Cardiol..

[13] Mora S, Glynn RJ, Ridker PM (2013). High-density lipoprotein cholesterol, size, particle number, and residual vascular risk after potent statin therapy. Circulation.

[14] Otvos JD (2011). Clinical implications of discordance between low-density lipoprotein cholesterol and particle number. J. Clin. Lipidol..

[15] Holmes MV (2018). Lipids, lipoproteins, and metabolites and risk of myocardial infarction and stroke. J. Am. Coll. Cardiol..

[16] Qi Y (2015). Cholesterol-overloaded HDL particles are independently associated with progression of carotid atherosclerosis in a cardiovascular disease-free population. J. Am. Coll. Cardiol..

[17] Martin SS (2015). HDL cholesterol subclasses, myocardial infarction, and mortality in secondary prevention: The lipoprotein investigators collaborative. Eur. Heart J..

[18] Albers JJ, Slee A, Fleg JL, O’Brien KD, Marcovina SM (2016). Relationship of baseline HDL subclasses, small dense LDL and LDL triglyceride to cardiovascular events in the AIM-HIGH clinical trial. Atherosclerosis.

[19] Kim DS (2014). HDL-3 is a superior predictor of carotid artery disease in a case-control cohort of 1725 participants. J. Am. Heart Assoc..

[20] Kim DS (2016). Concentration of smaller high-density lipoprotein particle (HDL-P) is inversely correlated with carotid intima media thickening after confounder adjustment: The multi ethnic study of atherosclerosis (MESA). J. Am. Heart Assoc..

[21] Tiozzo E (2014). High-density lipoprotein subfractions and carotid plaque: The Northern Manhattan Study. Atherosclerosis.

[22] Potočnjak I (2017). Serum concentration of HDL particles predicts mortality in acute heart failure patients. Sci. Rep..

[23] Degoricija V (2019). HDL subclasses and mortality in acute heart failure patients. Clin. Chim. Acta.

[24] Hunter WG (2019). High-density lipoprotein particle subfractions in heart failure with preserved or reduced ejection fraction. J. Am. Coll. Cardiol..

[25] Ponikowski P (2016). 2016 ESC Guidelines for the diagnosis and treatment of acute and chronic heart failure. Eur. Heart J..

[26] Gottdiener JS (2004). American Society of Echocardiography recommendations for use of echocardiography in clinical trials. J. Am. Soc. Echocardiogr..

[27] Lang RM (2015). Recommendations for cardiac chamber quantification by echocardiography in adults: An update from the American Society of Echocardiography and the European Association of Cardiovascular Imaging. Eur. Hear. J. Cardiovasc. Imaging.

[28] Association WM (2013). World medical association declaration of Helsinki. JAMA.

[29] Mallol R (2015). Liposcale: A novel advanced lipoprotein test based on 2D diffusion-ordered 1 H NMR spectroscopy. J. Lipid Res..

[30] Gornik O, Lauc G (2008). Glycosylation of serum proteins in inflammatory diseases. Dis. Mark..

[31] Fuertes-Martín R (2019). Glycoprotein A and B height-to-width ratios as obesity-independent novel biomarkers of low-grade chronic inflammation in women with polycystic ovary syndrome (PCOS). J. Proteome Res..

[32] Akinkuolie AO, Buring JE, Ridker PM, Mora S (2014). A novel protein glycan biomarker and future cardiovascular disease events. J. Am. Heart Assoc..

[33] McGarrah RW (2017). A novel protein glycan-derived inflammation biomarker independently predicts cardiovascular disease and modifies the association of HDL subclasses with mortality. Clin. Chem..

[34] Velavan P, Huan Loh P, Clark A, Cleland JGF (2007). The cholesterol paradox in heart failure. Congest. Hear. Fail..

[35] Rauchhaus M (2003). The relationship between cholesterol and survival in patients with chronic heart failure. J. Am. Coll. Cardiol..

[36] Amigó N (2016). Lipoprotein hydrophobic core lipids are partially extruded to surface in smaller HDL: "Herniated" HDL, a common feature in diabetes. Sci. Rep..

[37] Toth PP (2013). High-density lipoproteins: A consensus statement from the National Lipid Association. J. Clin. Lipidol..

