# Supplementary material for: Particle size and cholesterol content of circulating HDL correlate with cardiovascular death in chronic heart failure
Source: Sci Rep. 2021 Feb 4;11:3141. doi: 10.1038/s41598-021-82861-6 (PMC7862293; doi:10.1038/s41598-021-82861-6)
Supplement: Supplementary file 1 — Supplementary Information [file 41598_2021_82861_MOESM1_ESM.docx]

**SUPPLEMENTARY MATERIAL FILE**

**Title**

Particle size and cholesterol content of circulating HDL correlate with cardiovascular death in chronic heart failure

**Author’s names and institutions**

Teis A; Cediel G; Amigó N; Julve J; Aranyó J; Andrés J; Puig-Jové C; Castelblanco E; Gual-Capllonch F; Ferrer-Sistach E; Vallejo N; Juncà G; López-Ayerbe J; De Antonio M; Domingo M; Santiago-Vacas E; Codina P; Mauricio D; Lupón J; Alonso N; Bayes-Genis A

**SUPPLEMENTARY MATERIAL**

**Supp. Figure 1.-**  Distribution of HDL-C/P ratio and HDL-Sz in the overall cohort

**Figure S1 legend**: HDL-C/P ratio: Content of Cholesterol per HDL particle. HDL-Sz: Mean normalized HDL particle size. Nm: nanometers

**Supp. Table S1.-** Univariable and multivariable Cox Regression Analysis of the associations between HDL-Sz and HDL-C/P ratio with cardiovascular death according to NYHA functional classification.

|  | **HDL-Sz** | | **HDL-C/P ratio** | |
| --- | --- | --- | --- | --- |
|  | **HR (95% CI)** | **P value** | **HR (95% CI)** | **P value** |
| **NYHA functional class I-II** | | | | |
| Unadjusted | 1.28 (1.05-1.56) | 0.016 | 1.05 (1.01-1.08) | 0.005 |
| Model 1 | 1.21 (0.97-1.51) | 0.087 | 1.03 (0.99-1.01) | 0.119 |
| Model 2 | 1.15 (0.93-1.43) | 0.199 | 1.03 (0.97-1.09) | 0.280 |
| **NYHA functional class III-IV** | | | | |
| Unadjusted | 1.34 (1.05-1.70) | 0.020 | 1.07 (1.01-1.13) | 0.028 |
| Model 1 | 1.38 (1.07 – 1.78) | 0.012 | 1.06 (1.00-1.13) | 0.044 |
| Model 2 | 1.38 (1.07-1.78) | 0.012 | 1.09 (1.02-1.15) | 0.006 |

Hazard ratios for HDL-Sz reflect 1 SD change in a given measure.

**Model 1** Adjusted for age, sex, BMI, diabetes, arterial hypertension, vasculopathy, ischemic etiology, eGFR, Hemoglobin, NT-ProBNP, LDL-P and treatment with ACEi/ARB, Betablockers, statins and ICD.

**Model 2** Adjusted for Model 1 plus glycoprotein (Gly)-A, Gly-B and Gly-F.
